# Supplementary material for: Saccharomyces boulardii (CNCM I-745) improves intestinal damage in sepsis by remodeling intestinal flora structure
Source: Front Cell Infect Microbiol. 2025 Jul 31;15:1602792. doi: 10.3389/fcimb.2025.1602792 (PMC12350329; doi:10.3389/fcimb.2025.1602792)
Supplement: Supplementary file 1 [file DataSheet1.zip › summary/summary/8_Advanced_analyse/2_PICRUSt2_result/KEGG/Group.CW0_vs_AW0/PICRUSt2_KEGG_level3_Group.CW0_vs_AW0_diff.pdf]

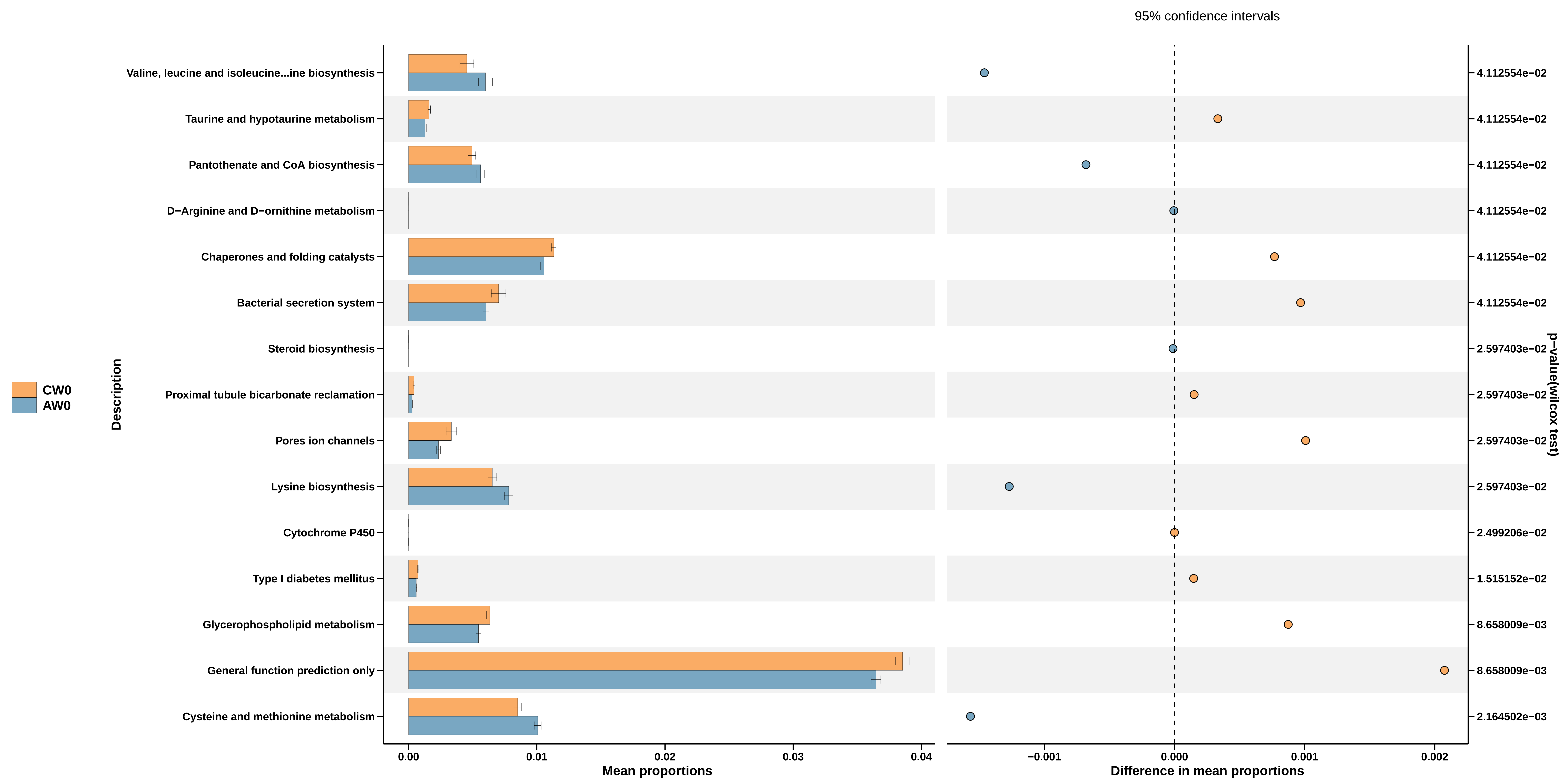
95% confidence intervals
Valine, leucine and isoleucine...ine biosynthesis
Taurine and hypotaurine metabolism
Pantothenate and CoA biosynthesis
D−Arginine and D−ornithine metabolism
Chaperones and folding catalysts
Bacterial secretion system
Steroid biosynthesis
Proximal tubule bicarbonate reclamation
Pores ion channels
Lysine biosynthesis
Cytochrome P450
Type I diabetes mellitus
Glycerophospholipid metabolism
General function prediction only
Cysteine and methionine metabolism
4.112554e−02
4.112554e−02
4.112554e−02
4.112554e−02
4.112554e−02
4.112554e−02
2.597403e−02
2.597403e−02
2.597403e−02
2.597403e−02
2.499206e−02
1.515152e−02
8.658009e−03
8.658009e−03
2.164502e−03
CW0
AW0
Description
0.00
0.01
0.02
0.03
0.04
Mean proportions
−0.001
0.000
0.001
0.002
Difference in mean proportions
p−value(wilcox test)
